# Alcohol Withdrawal Mimicking Neuroleptic Malignant Syndrome

**DOI:** 10.7759/cureus.4697

**Published:** 2019-05-18

**Authors:** Hafsa Farooq, Tayyaba Mohammad, Amna Farooq, Qasim Mohammad

**Affiliations:** 1 Internal Medicine, Waterbury Hospital, Waterbury, USA; 2 Internal Medicine, Rutgers New Jersey Medical School, Newark, USA; 3 Medicine, Shaikh Khalifa Bin Zayed Al-Nahyan Medical and Dental College, Lahore, PAK; 4 Internal Medicine, Windsor University School of Medicine, Boonton, USA

**Keywords:** alcohol withdrawal, neuroleptic malignant syndrome

## Abstract

Long-standing, heavy alcohol use can lead to alcohol dependence, which predisposes to alcohol withdrawal if alcohol consumption is suddenly decreased or stopped. Alcohol withdrawal syndrome is characterized by a hyperadrenergic response, with symptoms ranging from mild tremulousness to delirium tremens. We report a 55-year-old male presenting with hyperthermia, tachycardia, tachypnea, altered consciousness, tremors, rigidity, diaphoresis, elevated creatinine kinase, and myoglobinuria. The diagnosis of alcohol withdrawal was made due to a history of alcohol use disorder with the last drink two days ago and no history of any medication or drug intake prior to admission. He was treated with benzodiazepines with an improvement in his condition.

## Introduction

Alcohol withdrawal is a clinical syndrome that requires good history-taking and a thorough examination to support the diagnosis and rule out the other differentials. Clinical suspicion and prompt recognition of alcohol withdrawal syndrome are important for preventing the adverse effects related to delirium tremens due to the increased morbidity and mortality associated with it. We present a unique case of alcohol withdrawal whose presentation was similar to neuroleptic malignant syndrome but a detailed history and physical examination helped us make the definitive diagnosis. It is also very important to rule out other differentials, as a premature diagnosis of alcohol withdrawal can lead to the inappropriate use of sedatives.

## Case presentation

A 55-year-old male with a history of alcohol-use disorder presented with altered mental status and hyperthermia. The initial examination of the patient in the emergency department (ED) established hyperthermia (107.4°F), tachycardia (158 bpm), tachypnea (28/min), altered consciousness, generalized rigidity, tremors, diaphoresis, hyperactive bowel sounds, normal reflexes, clear lungs, acute respiratory alkalosis, anion gap metabolic acidosis with elevated lactic acid of 4.6 mmol/L (normal range 0.5-2.0 mmol/L), ethanol level of 30 mg/dL (normal <10 mg/dL), elevated creatinine kinase (CK) of 401 U/L (normal range 55-170 U/L) with myoglobinuria of 5060 mcg/L (normal <28 mcg/L), hyponatremia of 128 mmol/L (normal 136-146 mmol/L), hypomagnesemia of 0.6 mg/dL (normal 1.7-2.2 mg/dL), hypophosphatemia of 1.2 mg/dL (normal 2.5-4.5 mg/dL), elevated liver enzymes (aspartate aminotransferase (AST) 356 Int U/L > alanine aminotransferase (ALT) 98 Int U/L), normal leukocyte count of 9.8 thous/mm^3^, negative urine toxicology screen, and normal ammonia level of 15 mcmol/L (normal 9-33 mcmol/L). The patient was admitted to the intensive care unit (ICU) for the management of neuroleptic malignant syndrome based on his presentation and laboratory values. On further investigation, his computed tomography (CT) head was negative, chest X-ray was normal, and blood culture was negative. There was no history of any medication or drug intake as well. On further evaluation, it was found that the patient is a heavy alcohol user, with his last drink two days ago and his previous history of alcohol withdrawal had almost a similar presentation. His diagnosis was subsequently changed to a hyperadrenergic surge secondary to alcohol withdrawal instead of neuroleptic malignant syndrome. The patient was managed accordingly with benzodiazepines. His creatine kinase (CK) initially peaked to 1268 Int U/L on Day 2 and then down-trended to the normal level with hydration, along with a normalization of his serum electrolytes. The patient was provided counseling regarding abstinence from alcohol intake at the time of discharge.

## Discussion

Alcohol withdrawal syndrome is characterized by signs of the overactivity of the sympathetic nervous system. The physiology of an overactive sympathetic system in alcohol withdrawal is based on the upregulation of N-nitrosodimethylamine (NMDA) receptors. Alcohol is an agonist of gamma-aminobutyric acid (GABA) receptors and an antagonist of NDMA receptors. During repeated and prolonged use of alcohol, GABA receptors are downregulated and NDMA receptors are upregulated. In the absence of alcohol, the decreased number of GABA receptors and increased number of NDMA receptors lead to a synergistic adrenergic effect [1] and the severity of the symptoms correlates positively with the amount of adrenergic stimulation. The symptoms of alcohol withdrawal range from mild tremulousness to life-threatening delirium tremens (DT). DT can present as hallucinations, disorientation, tachycardia, hypertension, hyperthermia, agitation, diaphoresis, and possible seizures in the setting of acute reduction or abstinence from alcohol, which typically begins between 48 and 96 hours after the last drink and lasts one to five days [2-4]. Disorders such as neuroleptic malignant syndrome [5] and serotonin syndrome may have similar presentations and can be confused with DT or alcohol withdrawal. Alcohol withdrawal is purely a clinical diagnosis. A thorough history and physical is important in diagnosing the clinical syndrome as well as differentiating it from other possible diagnoses. It is also necessary to rule out other conditions, such as infection, trauma, metabolic derangements, drug overdose, and hepatic failure, particularly if the presentation includes altered mental status and fever. The treatment of choice for alcohol withdrawal is aimed at decreasing the hyperadrenergic response by the use of benzodiazepines.

## Conclusions

This case is presented to highlight the diagnosis of alcohol withdrawal. The presentation of alcohol withdrawal with an extreme adrenergic surge is rare, but it can be diagnosed with good history-taking and examination. Physicians should always consider the possibility of alcohol withdrawal in patients with an unusual presentation and a history of alcohol use.
